# Predictors and Recurrence Patterns After Radical Surgery in Ampulla of Vater Cancer: Comparative Analysis Between Early and Late Recurrence

**DOI:** 10.3389/fsurg.2022.833373

**Published:** 2022-03-18

**Authors:** Zheng-Yun Zhang, Da-Wei Liu, Di-Si Hao, Zun-Qiang Zhou

**Affiliations:** ^1^ Department of Surgery, Shanghai Jiao Tong University Affiliated Sixth People’s Hospital, Shanghai, China; ^2^ Department of Surgery, Heilongjiang Provincial Hospital Affiliated to Harbin Institute of Technology, Harbin, China

**Keywords:** adenocarcinoma, ampulla of Vater, predictors of recurrence, recurrence pattern, radical surgery

## Abstract

**Objective:**

Tumor recurrence remains the main dilemma after surgical treatment of ampulla of Vater carcinoma. This study was designed to identify the prognostic factors and evaluate the recurrence patterns of ampulla of Vater cancer.

**Methods:**

A total of 286 patients who underwent surgical resection of ampulla of Vater cancer in two medical centers from January 2000 to October 2016 were collected. Data on clinicopathologic factors, survival rate, and recurrence patterns were retrospectively analyzed.

**Results:**

A total of 158 patients (55.2%) survived without evidence of recurrence (non-recurrence), whereas 65 (22.7%) and 63 patients (22.1%) suffered from recurrence of the disease within 12 months (early recurrence) and after 12 months (late recurrence), respectively. Early-recurrence patients exhibited a more advanced disease (advanced tumor stage, lymph node involvement, pancreas invasion, and late TNM stage) than late-recurrence patients. The first or primary location of cancer recurrence in 33 patients (25.8%) was locoregional. Metastasis developed in the liver in 30 patients (23.4%), peritoneum in 13 patients (10.2%), lungs in 10 patients (7.8%), and para-aortic or superior mesenteric artery lymph node in 10 patients (7.8%). Multiple metastases were observed in 26 patients (20.3%).

**Conclusion:**

The most common patterns of postoperative recurrence are locoregional and recurrent liver metastasis. The recurrence patterns with the worst prognosis are peritoneal and multiple metastases.

## Introduction

Carcinoma of the ampulla of Vater has a relatively higher resection rate and more favorable prognosis than other malignant tumors of the periampullary region. The 5-year survival rate of ampulla of Vater cancer after radical surgery ranges from 35.0% to 62.7% (1–4). However, tumor recurrence frequently remains the main problem after surgical treatment of ampulla of Vater carcinoma, and recurrences are often found in various forms and at more than one site simultaneously. Therefore, confirming the patterns and evaluating the risk factors of recurrence are difficult. Numerous studies have investigated the clinicopathologic aspects of ampulla of Vater carcinoma, but most have focused only on the prognosis or survival of the disease (5, 6). An examination of the predictors and recurrence patterns can establish the criteria for postoperative adjuvant therapy, which can postpone recurrence and improve survival (5, 7, 8). In the present study, patterns of relapses were investigated, and the correlation between histopathologic factors and recurrence was evaluated to provide a basis for the development of further therapy.

## Methods

### Patients

The findings on 328 consecutive patients who were operated for ampulla of Vater adenocarcinoma from January 2000 to October 2016 in Shanghai Jiao Tong University Affiliated Sixth People’s Hospital and Heilongjiang Provincial Hospital Affiliated to Harbin Institute of Technology were retrospectively reviewed. Forty-two patients who underwent palliative operation were excluded. A total of 286 patients underwent pancreaticoduodenectomy with regional lymphadenectomy. We routinely performed lymph node dissection in the hilar region, the upper and lower edges of the pancreas, and around the celiac trunk vessels, without cleaning the lymph nodes near the inferior vena cava. The median number of lymph node dissection was 23 (16–44). Rapid frozen pathological examination was performed routinely during the operation, and the benign and malignant tumors of the cutting edge were determined. The medical records of all 286 patients were retrospectively reviewed for demographics, laboratory findings on admission and postoperation, types of procedure, operative findings, and histopathologic findings. Percutaneous transhepatic biliary drainage or endoscopic nasobiliary drainage was routinely used to relieve obstructive jaundice before operation if the bilirubin level was higher than 10 mg/dL.The type of resection, either Whipple or pylorus-preserving pancreaticoduodenectomy, was based on the surgeon’s judgment. Perioperative and follow-up details were compared among patients with tumor recurrence and non-recurrence and early and late recurrence. The time from operation to recurrence varied (1–78 months). The median time of recurrence was 11 months. For the purpose of data analysis, patients with recurrence were therefore operationally categorized into early- or late-recurrence groups with a cutoff time point of 12 months. This study was conducted according to the principles outlined in the Declaration of Helsinki, and patients provided written informed consent.

### Histopathology

All tumors were adenocarcinomas that originated in the ampulla of Vater. Histologic differentiation was recorded as well, moderately, or poorly differentiated. Tumor size was measured from surgical specimens before formalin fixation. The tumor and TNM stages were defined according to the American Joint Committee on Cancer (AJCC, 8th Edition) classification of 2016 (9). The resection margin was evaluated by a pathologist. R0 resection indicated that the resection margins of the pancreas, common bile duct, duodenum, and retropancreatic tissue were histologically free of carcinoma. R1 resection indicated histologically positive for carcinoma cells in the resection margin, but grossly free, based on the surgeon’s judgement (10–12).

### Definition of complications and follow-up

Follow-up at 3-month intervals comprised physical examination, laboratory tests, and tumor markers (CA199). Abdominal computed tomography was arranged every 3 months in the first year and then every 6 months in the second year. Radiography of the thorax, bone scan, and computed tomography of the brain were performed if clinical examination led to a suspicion of metastasis. Disease relapse was defined as a biopsy-proven disease or radiologic evidence of recurrence. Recurrence was categorized as locoregional or metastatic. Local recurrence was defined as a recurrent retroperitoneal mass or regional node, and metastasis was defined as a relapse of the disease at a distant site, either a visceral organ or a non-regional lymph node. Multiple metastases were defined as more than one site of metastases. The primary endpoint of the study was death, and the primary criterion of follow-up was survival time.

### Statistical analysis

Continuous variables were expressed as median and range or mean ± standard deviation, while categorical variables were reported as number and percentage. Chi-square test was used for nominal data, and univariate analysis was performed with χ^2^ test or Fisher’s exact test for categorical variables. Comparison between the early- and late-recurrence groups was separately performed. For those that did not follow normal distributions, non-parametric Mann–Whitney U test was used. Disease-free survival was estimated with the Kaplan–Meier method and compared with log-rank test. Disease-free survival was measured from the date of surgery to the date of recurrence or last contact. Factors found to be statistically significant (p < 0.1) by univariate analysis were subjected to multivariate analysis with Cox proportional-hazard regression using forward stepwise procedure (13). Data were considered significant at p < 0.05. SPSS 20.0 statistical software (SPSS, Chicago, IL) was used for all statistical analyses.

## Results

### Demographics

A total of 286 patients were enrolled in the study. The non-recurrence group consisted of 158 patients (55.2%), the early-recurrence group consisted of 65 patients (22.7%), and the late-recurrence group consisted of 63 patients (22.1%). No statistical difference was observed between the non-recurrence and recurrence groups in terms of age, gender, body mass index (BMI), total bilirubin, or operative methods. The median follow-up periods in the non-recurrence and recurrence groups were 59 months (range: 1 to 178 months) and 31.5 months (range: 2 to 111 months), respectively. The median times from surgery to recurrence in the early- and late-recurrence groups were 6 months (range: 1 to 11 months) and 30 months (range: 12 to 78 months), respectively. The non-recurrence group had a lower preoperative or postoperative CA199 level than the recurrence group (p < 0.01) (Table 1). To focus on distinctions between early and late recurrence, the patients’ demographics are also shown in Table 1. The early-recurrence group had a higher preoperative CA199 level than the late-recurrence group (p < 0.05). Early-recurrence patients did not have a significantly higher post or recurrent CA199 level than late-recurrence patients. No statistical significance was observed between the early- and late-recurrence groups in terms of age, gender, BMI, total bilirubin, or operative methods. A total of 89 patients (31.1%) received postoperative adjuvant chemotherapy. No patients received neoadjuvant or intraoperative radiation therapy. Concurrent chemotherapy for most patients included continuous infusion of 5-fluorouracil and gemcitabine.

**Table 1:** Demographics of patients with ampullary cancer who underwent radical resection.

Demographics	Non-recurrence	Recurrence	P value	Early recurrence	Late recurrence	P value
Patients, n (%)	158 (55.2%)	128 (44.8%)		65 (22.7%)	63 (22.1%)	
Age, mean + SD	65.1 ± 11.1	66.2 ± 11.4	NS	64.7 ± 11.4	67.8 ± 11.1	NS
Gender, (F/M)	74/84	53/75	NS	21/44	32/31	NS
BMI, mean + SD	23.5 ± 2.8	23.1 ± 3.4	NS	22.7 ± 3.6	23.5 ± 3.2	NS
Total bilirubin (mg/dL)	6.1 (0.5-43.1)	4.8 (0.6-26.6)	NS	7.4 ± 6.2	5.6 ± 5.5	NS
Operative procedure, n (%)			NS			NS
Whipple	25	28		16	12	
PPPD	133	100		49	51	
Adjuvant chemotherapy	20	69	<0.001	35	34	NS
CA199 (pre-operation) (U/dL)	134.0 (6.0-4480.0)	264.5 (1.5-10900.0)	<0.01	57.6 (1.5-1800.0)	32.6 (2.6-10900.0)	<0.05
CA199 (1 month post-operation) (U/dL)	8.9 (0.6-53.7)	246.0 (0.3-20600.0)	<0.01	14.3 (0.3-20600.0)	10.7 (1.6-1883.0)	NS
CA199 (Recurrence) (U/dL)		71.3 (1.0-41900.0)		78.1 (51.0-41900.0)	74.5 (43.0-42500.0)	NS
Follow-up (months, median[range])	59.0 (1.0-178.0)	31.5 (2.0-111.0)		16.0 (2.0-47.0)	46.0 (15.0-111.0)	

*NS: not significant*.

### Histopathologic findings

The results of univariate analysis of risk factors and histopathologic results between the non-recurrence and recurrence groups and the early- and late-recurrence groups are shown in Table 2. No correlation in tumor size was observed among the four groups. With regard to tumor stage, 3.1% of patients in the early-recurrence group had a T1 lesion, while 96.9% had T2, T3, or T4 lesions; meanwhile, 85.7% and 71.5% of patients in the late- and non-recurrence groups had T2, T3, or T4 lesions, respectively. A total of 56 patients (43.8%) and 37 patients (23.4%) had pancreatic invasion (T3) in the recurrence and non-recurrence groups, respectively. In the recurrence group, 34 patients (52.3%) and 22 patients (34.9%) had pancreatic invasion in the early-recurrence and late-recurrence groups, respectively. All patients in the non-recurrence groups had complete resection (R0 resection); however, three and two patients in the early-recurrence and late-recurrence groups had residual microscopic disease (R1 resection), respectively (p > 0.05). No significant difference in the number of total dissected lymph nodes was observed among the four groups. The ratio of lymph node involvement was 19.6% and 39.8% in the non-recurrence and recurrence groups (p < 0.001) and 50.8% and 30.2% in the early- and late-recurrence groups, respectively (p < 0.05). The ratio of lymphovascular invasion in the non-recurrence group was 24.1%, which was significantly lower than that in the recurrence group (39.1%) (p < 0.001). In the early-recurrence group, the ratio of lymphovascular invasion was 50.8%, which was higher than that in the late-recurrence group (27%) (p < 0.01). The ratio of perineural invasion in the non-recurrence group was lower than that in the recurrence group (p < 0.001), but no statistical significance was observed between the early- and late-recurrence groups. Furthermore, the ratios of moderate and poor differentiation were higher in the early-recurrence group (72.3% and 20% compared with 60.3% and 4.8% in the late-recurrence group, p < 0.01). The non-recurrence patients had the disease at an early stage (51.3% in stage I, 31.0% in stage II, and 17.7% in stage III), whereas the recurrence patients had the disease at an advanced stage (28.1% in stage I, 50% in stage II, and 21.9% in stage III, p < 0.001). The early-recurrence patients had a more advanced disease (16.9% in stage I, 58.5% in stage II, and 24.6% in stage III) than late-recurrence ones (39.7% in stage I, 41.3% in stage II, and 19% in stage III, p < 0.05).

**Table 2:** Histopathologic findings in patients: results of univariate analysis.

	Non-recurrence	Recurrence	P value	Early- recurrence	Late- recurrence	P value
Patients, n (%)	158 (55.2%)	128 (44.8%)		65 (22.7%)	63 (22.1%)	
Tumor size (cm)	2.2 ± 1.2	2.4 ± 1.3	NS	2.6 ± 1.3	2.1 ± 1.2	NS
Tumor stage, n (%)						
T1	45 (28.5%)	11 (8.6%)	<0.001	2 (3.1%)	9 (14.3%)	<0.001
T2 (duodenal invasion)	48 (30.4%)	35 (27.3%)	NS	15 (23.1%)	20 (31.7%)	NS
T3 (pancreas invasion)	37 (23.4%)	56 (43.8%)	<0.001	34 (52.3%)	22 (34.9%)	<0.05
T4	28 (17.7%)	26 (20.3%)		14 (21.5%)	12 (19.0%)	
Resection margin, n (%)			NS			NS
R0	158	123		62	61	
R1	0	5		3	2	
R2	0	0		0	0	
Resected lymph node (median[range])	9 (0-50)	9 (0-55)	NS	10 (0-55)	9 (0-24)	NS
Positive lymph node (median[range])	4 (0-9)	6 (0-14)	<0.001	6 (0-14)	7 (0-11)	<0.01
Lymph node status, n (%)			<0.001			<0.05
Negative	127 (80.4%)	77 (60.2%)		32 (49.2%)	44 (69.8%)	
Positive	31 (19.6%)	51 (39.8%)		33 (50.8%)	19 (30.2%)	
Lymphovascular invasion			<0.001			<0.01
Negative	120 (75.9%)	78 (60.9%)		32 (49.2%)	46 (73.0%)	
Positive	38 (24.1%)	50 (39.1%)		33 (50.8%)	17 (27.0%)	
Perineural invasion			<0.001			NS
Negative	138 (87.3%)	92 (71.9%)		42 (64.6%)	50 (79.4%)	
Positive	20 (12.7%)	36 (28.1%)		23 (35.4%)	13 (20.6%)	
Differentiation, n (%)			<0.001			<0.001
Well	79 (50.0%)	27 (21.1%)		5 (7.7%)	22 (34.9%)	
Moderate	71 (44.9%)	85 (66.4%)		47 (72.3%)	38 (60.3%)	
Poor	8 (5.1%)	16 (12.5%)		13 (20.0%)	3 (4.8%)	
AJCC TNM Stage, n (%)			<0.001			<0.05
Stage I	81 (51.3%)	36 (28.1%)		11 (16.9%)	25 (39.7%)	
Stage II	49 (31.1%)	64 (50.0%)		38 (58.5%)	26 (41.3%)	
Stage III	28 (17.7%)	28 (21.9%)		16 (24.6%)	12 (19.0%)	

*NS: not significant*.

### Recurrence patterns

A total of 158 patients (55.2%) in the non-recurrence group had no recurrent disease during follow-up. Cancer recurrence was observed in 128 (44.8%) patients, and the first or primary location of cancer recurrence was locoregional in 33 patients (25.8%). Metastasis developed in the liver in 30 patients (23.4%), peritoneum in 13 patients (10.2%), lungs in 10 patients (7.8%), and para-aortic or superior mesenteric artery (SMA) lymph node in 10 patients (7.8%). Multiple metastases were observed in 26 patients (20.3%). The recurrence of other diseases (e.g., bone metastasis and Virchow’s lymph node) was observed in six patients (4.7%). To focus on distinctions between early and late recurrence, the details of recurrence patterns are shown in Table 3. The recurrence patterns were similar between the early- and late-recurrence groups.

**Table 3:** Recurrence patterns in 128 patients with ampullary of vater cancer who underwent radical surgery, comparing early and late recurrence group.

	Early recurrence	Late recurrence	P value
Local recurrence	13 (20%)	20 (31.8%)	NS
Metastasis	34 (52.3%)	29 (46.0%)	NS
Liver metastasis	18	12	NS
Peritoneal carcinomatosis	9	4	NS
Lung metastasis	3	7	NS
Paraaortic/SMA LN	4	6	NS
Multiple metastasis	16 (24.6%)	10 (15.9%)	NS
Others*	2 (3.1%)	4 (6.3%)	NS

*Others including bone, colon and Virchow’s lymph node metastases, NS: not significant.

### Patients’ survival

**Figure 1:**
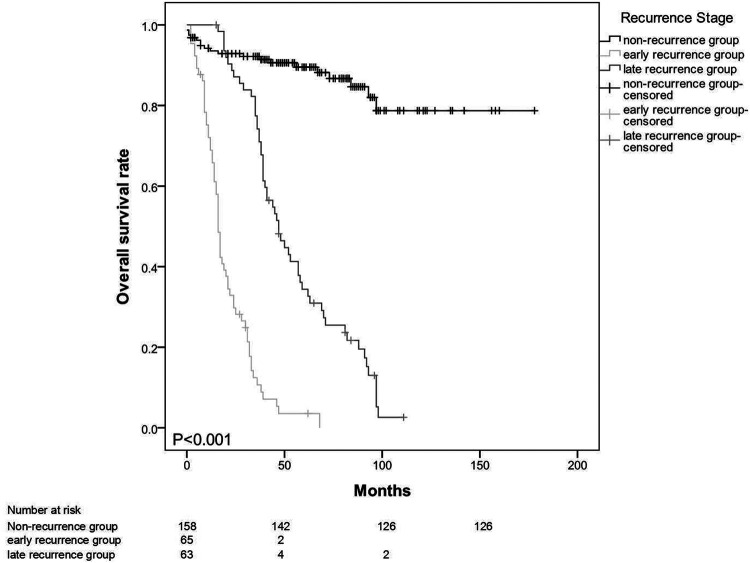
Median survival time (from operation to death) in the early and late-recurrence groups at 16 (2-68) months vs 46 (15-111) months (p < 0.001).

**Figure 2:**
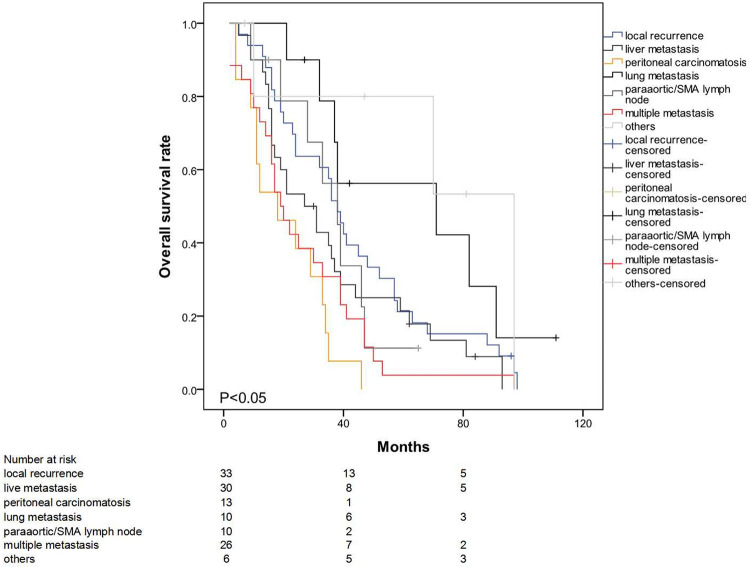
Significant effect of peritoneal seeding, para-aortic/SMA lymph node metastasis and multiple metastasis on survival (p < 0.05).

The mean survival time for the entire patient population was 91.7 months (95% CI: 81.8–101.7). The estimated overall survival rates at 3 and 5 years were 69.4% and 55.5%, respectively. The median survival times (from operation to death) in the early- and late-recurrence groups were 16 (2–68) and 46 (15–11) months (p < 0.001) (Figure 1). The patterns of the recurrence group showed that peritoneal seeding, para-aortic/SMA lymph node metastasis, and multiple metastases significantly influenced survival as indicated in the univariate analysis (p < 0.05) (Figure 2). The median time from disease recurrence to death was 9 (0–57) and 16 (0–76) months in the early- and late-recurrence groups (p > 0.05), respectively. The mean survival times of patients with peritoneal seeding and multiple metastases were shorter than those of others at 20.8 and 26.4 months, respectively. Eighty-nine patients (31.1%) received postoperative adjuvant chemotherapy. Seventeen patients with chemotherapy for local recurrence showed an improved survival rate compared with 16 patients without chemotherapy.

In the univariate analysis, tumor stage, resection margin, lymph node involvement, lymphovascular invasion, perineural invasion, tumor differentiation, AJCC TNM stage, and chemotherapy were the significant predictors of recurrence (Table 4). In multivariate analysis, histologic differentiation (RR = 2.4, 95% CI: 1.9–3.9, p < 0.01), lymphovascular invasion (RR = 2.2, 95% CI: 1.5–3.0, p < 0.01), peritoneal seeding (RR = 12.2, 95% CI: 5.6–43.8, p < 0.01), and multiple metastases (RR = 6.7, 95% CI: 2.4–18.3, p < 0.01) were the significant predictors of early recurrence. Lymphovascular invasion (RR = 2.5, 95% CI: 1.2–3.7, p < 0.01), peritoneal seeding (RR = 6.9, 95% CI: 3.5–9.6, p < 0.01), and multiple metastases (RR = 5.4, 95% CI: 2.1–7.8, p < 0.01) were the significant predictors of late recurrence (Table 5). Further analysis showed that the majority of the patients in early recurrence group had moderate (72.3%) and poor (20.0%) differentiation lesions (Table 2).

**Table 4:** Variables on disease-specific survival in 286 patients with ampullary of vater cancer who underwent radical surgery.

Variables	No. patients	Mean survival time (months)	Survival rate (%)		P value
			3-year	5-year	
Tumor stage, n (%)					<0.001
T1	56	88.7	92.3	75.8	
T2	83	92.5	76.6	65.7	
T3	93	68.2	52.5	35.9	
T4	54	58.1	51.0	35.7	
Resection margin, n (%)					<0.01
R0	281	92.9	70.0	55.9	
R1	5	33.0	30.0	0.0	
Lymph node status, n (%)					<0.001
Negative	204	98.6	78.7	62.4	
Positive	82	68.1	50.0	38.0	
Lymphovascular invasion					<0.001
Negative	198	93.4	76.6	61.4	
Positive	88	73.5	51.9	41.3	
Perineural invasion					<0.001
Negative	230	100.6	75.4	61.0	
Positive	56	43.8	47.5	30.2	
Differentiation, n (%)					<0.001
Well	106	119.2	87.5	77.5	
Moderate	156	71.5	62.7	44.2	
Poor	24	49.1	43.3	40.5	
AJCC TNM Stage, n (%)					<0.001
Stage I	117	94.8	86.8	72.1	
Stage II	113	73.9	60.5	39.9	
Stage III	56	64.3	56.8	31.7	
Chemotherapy					<0.001
Yes	89	42.6	44.5	26.3	
No	197	106.6	75.6	61.4	

**Table 5:** Multivariate analysis in the recurrence group.

Group	Variables	Relative risk	95% Confidence interval	P value
Early recurrence	Histologic differentiation	2.4	1.9-3.9	<0.01
	Lymphovascular invasion	2.2	1.5-3.0	<0.01
	Peritoneal seeding	12.2	5.6-43.8	<0.01
	Multiple metastasis	6.7	2.4-18.3	<0.01
Late recurrence	Lymphovascular invasion	2.5	1.2-3.7	<0.01
	Peritoneal seeding	6.9	3.5-9.6	<0.01
	Multiple metastasis	5.4	2.1-7.8	<0.01

## Discussion

Carcinoma of the ampulla of Vater is a rare neoplasm, but has a relatively favorable prognosis compared with other periampullary neoplasms, such as those of the pancreatic head or distal bile duct (12, 14, 15). The difficulties associated with surgery include higher frequencies of regional or distant lymph node metastases and vascular involvement, and positive resection margins in pancreatic and retroperitoneal tissues. Radical resections increase margin negativity and life expectancy; however, the extend of the surgery applied is controversial. Thus, western and eastern centers may use different approaches (16). Nevertheless, similar to other malignancies of this region, tumor recurrence after radical resection of ampulla of Vater carcinoma eventually leads to the death of the patient (17). In the literature, several risk factors for ampullary cancer have been identified, including tumor size, histologic grade, invasion depth, tumor morphology, lymph node involvement, blood transfusion, and positive resection margin (18, 19). In this study, lymph node involvement, lymphovascular invasion, histologic differentiation, perineural invasion, and AJCC TNM stage were identified as the significant predictors of recurrence.

Lymph node involvement is a significant predictor of poor prognosis (20). In this study, regional lymphadenectomy, not including para-aortic and distant nodes as in radical lymphadenectomy, was performed. The ratio of lymph node involvement was 39.8% in the recurrence group, 50.8% in the early-recurrence group, and 30.2% in the late-recurrence group (p < 0.05). Further comparisons between early- and late-recurrence groups showed that the effects of lymph node involvement and tumor differentiation were significant. Lymph node involvement can indicate more advanced disease characteristics.

The 5-year survival rate in patients with lymph node involvement ranged from 0% to 41% and that in patients without it ranged from 67% to 81% (20). Our series showed similar results: 82 patients with lymph node involvement had a significantly shorter mean survival time (68.1 months) and 5-year survival (38.0%) than patients without lymph node involvement (5-year survival: 62.4%).

Klempnauer et al. identified the determinants of long-term survival after resection of ampullary cancer by comparing short- and long-term survivors (21). They found that tumor size, lymph node metastasis, Union for International Cancer Control stage, and grading significantly affect long-term survival, but only tumor size showed independent prognostic significance in multivariate analysis. Delcore et al. reported that tumor size was one of the independent factors for recurrence and 5-year survival (22). By contrast, Begar et al. showed that pancreatic invasion, not tumor size, was the pivotal factor for survival and recurrence (23). The present study obtained similar results: The number of patients with pancreatic invasion in the recurrence group (n = 56, 43.8%) was higher than that in the non-recurrence group (n = 37, 23.4%) (p < 0.001). Once the tumors penetrate the sphincter of Oddi, ampullary cancer causes nodal involvement and recurrences. The depth of invasion is a significant parameter of the degree of tumor spread and prognosis (4, 24). From the anatomical character of the ampulla of Vater, the pancreatic side of the duodenum has the lymphovascular supplement of the periampullary region. If the large tumor grows into the duodenal lumen but not into the pancreas, subsequent metastasis will not develop. Therefore, the negative effect should come from pancreatic invasion, not the large tumor size. The disease-free and overall survival in patients with intestinal subtype was superior to that in patients with pancreaticobiliary subtype.

Several reports showed through univariate analysis that tumor differentiation was also a predictor of survival, but the same was not the case when multivariate analysis was used. The 5-year survival rate was reported at 82% in patients with a well-differentiated tumor and 37% in patients with a moderately to poorly differentiated tumor. In this series, the 5-year survival rates were dismal in those with a well-differentiated and moderately or poorly differentiated tumor. However, the differentiation had no influence on liver metastasis or local recurrence.

The TNM staging system is a popular predictor of survival, but survival rates vary in different reports. In some reports, the 5-year survival rate was 76%–100% for patients with stage I disease, 21%–70% for stage II disease, 10%–27% for stage III disease, and 0% for stage IV disease. In this series, the 5-year survival rate was 72.1% for patients with stage I disease, 39.9% for stage II disease, and 31.7% for stage III disease (25, 26).

Very few previous studies have focused on the recurrence patterns of ampulla of Vater carcinoma after surgical resection, even if these significantly affect patients’ survival. The most common pattern of relapse in ampullary cancer is liver metastasis, which ranges from 53%–67%, followed by distant lymph nodes (28%–60%), peritoneal carcinomatosis (20%–21%), lungs (21%–22%), and bone metastases (13%–17%). The incidence of locoregional recurrence is variable from 33% to 60%, but it is usually combined with distant metastasis (50%–100%) (16, 27).

In the present study, a total of 128 patients (44.8%) developed disease relapse, with 26 patients having more than one site of metastases. Thirty-three patients (25.8%) suffered from locoregional recurrence, while 30 patients (23.4%) had liver metastasis. Other sites of metastases included peritoneal carcinomatosis (13 cases), lungs (10 cases, 7.8%), and para-aortic or SMA lymph node (10 cases, 7.8%). The recurrence patterns were similar in the early- and late-recurrence groups.

Reports on the use of adjuvant therapy following radical resection of ampullary carcinoma are rare (28). Bakkevold et al. found that combined chemotherapy regimens can delay the incidence of recurrence in the first 2 years, but an increase in cure rate was not observed (29). Furthermore, a combination of intraoperative radiation and resection did not benefit patients with ampullary cancer. However, Lee and Willett et al. found that the combination of adjuvant chemotherapy and radiation therapy resulted in good local control in high-risk patients, but no improvement in survival (30–32). Mehta et al. proved that adjuvant chemoradiotherapy for ampullary cancer was well tolerated (33), but Sikora et al. found no improvement in long-term survival and no decrease in recurrence rates (34). Earlier studies indicated no known proper and adequate regimen of chemoradiotherapy for patients with ampullary cancer. Therefore, a prospective randomized study based on histopathologic or clinical predictors is necessary to elucidate the effect of adjuvant therapy on high-risk patients. Based on the results of pancreatic cancer studies, several institutions have extrapolated the incorporation of adjuvant therapy in patients with ampullary cancer. However, the use of adjuvant therapy in this setting is controversial. The results of a 1999 trial of the European Organization for Research and Treatment of Cancer demonstrated no statistically significant benefit in survival with adjuvant chemotherapy following resection for ampullary adenocarcinoma. In the present study, patients with resected ampullary adenocarcinoma do appear to benefit from adjuvant chemotherapy.

This study had some limitations concerning its retrospective nature. Though there were limitations in this study, the differences between groups were remarkable. In conclusion, lymph node involvement, lymphovascular invasion, peritoneal metastases, histologic differentiation, and AJCC TNM stage were significant predictors of recurrence. Lymphovascular invasion and histologic differentiation were significant histologic predictors of survival in patients with ampullary cancer. The most common patterns of postoperative recurrence are locoregional and recurrent liver metastasis. The recurrence patterns with the worst prognosis were peritoneal and multiple metastases. Postoperative regional therapy is recommended for patients with pancreatic invasion to decrease locoregional recurrence.

## Data Availability

The raw data supporting the conclusions of this article will be made available by the authors, without undue reservation.
